# Neuroprotective, Antioxidant and Anti-Inflammatory Effect of Greek Pomegranate Seed Oil on N2a Neuroblastoma Cells and Mild Cognitive Impairment Patients

**DOI:** 10.3390/biology14050548

**Published:** 2025-05-15

**Authors:** Eleni E. Tzekaki, Georgios Katsipis, Athanasios Chatzikostopoulos, Anna Koutoupa, Sophia N. Lavrentiadou, Magda Tsolaki, Anastasia A. Pantazaki

**Affiliations:** 1 Laboratory of Biochemistry, Department of Chemistry, Aristotle University of Thessaloniki, 54124 Thessaloniki, Greece; etzekaki@chem.auth.gr (E.E.T.); gkatsipis@chem.auth.gr (G.K.); 2 Center for Interdisciplinary Research and Innovation, Laboratory of Neurodegenerative Diseases (LND), Thermi, 57001 Thessaloniki, Greece; thachatziko@gmail.com (A.C.); slavrent@vet.auth.gr (S.N.L.); tsolakim1@gmail.com (M.T.); 3 Greek Association of Alzheimer’s Disease and Related Disorders (GAADRD), 54643 Thessaloniki, Greece; akoutoupa@gmail.com; 4 Laboratory of Animal Physiology, School of Veterinary Medicine, Aristotle University of Thessaloniki, Thessaloniki 54124, Greece

**Keywords:** mild cognitive impairment, Alzheimer’s disease, pomegranate seed oil, neuroinflammation, antioxidant therapy

## Abstract

Alzheimer’s disease (AD) remains a significant global health challenge with limited FDA-approved treatments, necessitating the search for novel preventive strategies. Antioxidants that are present in fruits and vegetables have garnered attention due to their potential neuroprotective effects. Among these, pomegranate (*Punica granatum* L.) has emerged as a promising source of neuroprotective antioxidants as it is rich in polyphenols, flavonoids, and hydrolysable tannins. Pomegranate seed oil (PSO) is a source of bioactive compounds that may modulate key pathological processes of AD. This study investigated the therapeutic potential of PSO in murine neuroblastoma N2a cells treated with lipopolysaccharide (LPS) to simulate AD-like inflammation. The effects of PSO on inflammation and oxidative stress markers, including TNF-α, iNOS, SOD1, and IL1β, were evaluated, along with changes in AD-related biomarkers Aβ_42_, Aβ_40_, and p-tau181. Additionally, the study extended its findings to clinical settings by assessing the impact of supervised PSO consumption for 12 months on similar biomarkers in patients with mild cognitive impairment. Results from this integrative approach demonstrated the anti-inflammatory and antioxidant potential of PSO, supporting its role in modulating AD-associated pathophysiology. These findings suggest that PSO may serve as an early-stage intervention to delay or mitigate AD progression, highlighting its therapeutic potential in preclinical and clinical contexts.

## Simple Summary

Alzheimer’s disease is a serious brain condition that causes memory loss for which, currently, very few treatment options exist. Scientists are searching for new ways to slow or prevent its progress, and natural substances present in fruits and vegetables are being studied for their protective effects on the brain. Pomegranate seed oil, extracted from the seeds of the fruit, contains natural compounds that may help reduce brain inflammation and damage. This study tested the effects of pomegranate seed oil on brain cells exposed to bacterial substances that mimic Alzheimer’s-like inflammatory phenomena. The oil appeared to reduce harmful inflammation and protect against cell damage. In addition, studies in humans have shown that consumption of pomegranate seed oil for one year had similar beneficial effect on people with mild cognitive problems. These findings suggest that pomegranate seed oil may help slow the early stages of Alzheimer’s disease. This research is important because it offers hope for a natural and accessible way to protect brain health and improve quality of life for people at risk of developing this condition.

## 1. Introduction

Alzheimer’s disease (AD) is the most prevalent form of dementia and the top cause of elderly disability worldwide. Healthcare costs for AD management have reached unprecedented heights, and there is still no convincing and safe pharmacological treatment for stalling the disease progression [1]. The progression of AD follows three phases. The first phase is presymptomatic, also known as “normal condition”, where there is no cognitive impairment. The MCI stage can last from two to seven years [2]. Progression to clinically diagnosable AD (third phase) occurs at a higher rate from MCI than from normal cognition, with an average rate of 10–15% per year [3].

The pathological characteristics of AD in the brain include the accumulation of beta-amyloid (Aβ) plaques and neurofibrillary tangles (NFTs) composed of tau protein, leading to neuronal and synaptic loss and subsequent cognitive decline [4,5]. Aβ peptides (mainly Aβ_40_ and Aβ_42_,—consisting of 40 and 42 amino acids, respectively) are derived from the stepwise enzymatic cleavage of the amyloid precursor protein (APP) by beta- and gamma-secretase. On the other hand, NFTs are a mixture of hyperphosphorylated forms of tau on different sites [6]. In the presence of these pathological aggregates, aged microglial cells acquire an over-activated phenotype, thus releasing pro-inflammatory cytokines such as interleukin 1β (IL-1β), tumor necrosis factor-alpha (TNF-α), and IL-6 [7,8,9]. Aβ aggregates can chemotactically attract more microglial cells and astrocytes to the site of accumulated Aβ, thus leading to a widespread, chronic neuroinflammatory condition in the brain [10].

Growing evidence indicates that oxidative stress, commonly linked to aging, is a significant and early hallmark of AD and contributes to its development [11]. Brain tissue contains various sources of reactive oxygen species (ROS) and exhibits a high oxidative capacity; however, its ability to counteract oxidative stress is limited. Aβ and tau accumulations can induce ROS overproduction from glial cells and mitochondrial dysfunction, further leading to neurodegenerative phenomena, including synaptic loss, vascular dysfunction, cholinergic denervation, neurotransmitter imbalance, and neuronal damage. In addition, mitochondrial dysfunction in AD contributes to excitotoxicity, disrupted ATP generation, and calcium imbalance, highlighting oxidative stress as a central factor in AD pathophysiology [12]. Furthermore, the brain is rich in peroxidation-susceptible lipids. Malondialdehyde (MDA)—a lipid peroxidation end product was previously found to increase in the brain and blood of MCI and AD patients [13,14]. Additionally, superoxide dismutase (SOD) is one of the first antioxidant defenses against oxidative stress. MnSOD (SOD2), eliminates O2^•−^ formed in the matrix or on the inner side of the inner membrane, while copper-zinc SOD (SOD1) is located and activated in the cytoplasm of eukaryotic cells [15]. Total SOD activity is found to increase in the brains of patients with AD [16].

The approved therapeutic interventions for AD are scarce, and the results in most cases are uncertain and possibly with side effects [17]. In addition, early prevention is of grave importance, as research and clinical data suggest that possible treatment is viable only at the early stages, as in the MCI stage [3]. Hence, researchers endeavor to uncover new preventive vehicles to delay AD [18]. Daily consumption of antioxidants found in nutritious foods such as fruits, vegetables, and nuts, is postulated to improve brain function and promote healthy aging [19,20]. Pomegranate (*Punica granatum* L.) has gained attention as a natural remedy due to its rich antioxidant content and neuroprotective properties [21,22,23]. Known for its diverse nutraceutical profile, the fruit’s non-edible parts, such as peels and seeds, are particularly rich in bioactive compounds, including polyphenols, flavonoids, and tannins, which contribute to its antioxidant, anti-inflammatory, and cardioprotective effects. Additionally, its array of vitamins (B, C, E, and K), microelements, and anthocyanins enhances its therapeutic potential across various systems and health conditions [24,25].

Pomegranate extracts from peels and seeds have demonstrated potent antioxidant effects. Recent studies and reviews emphasize the curative potential of phenolic compounds found in pomegranate by-products [26]. Research conducted through in vitro and in vivo models consistently highlights the significant antioxidant activity of pomegranate peels, seeds, and juice [27,28,29,30]. Moreover, studies have highlighted PF’s potential role in combating AD by attenuating neuroinflammation [31] or reverse amyloidosis, a hallmark of AD pathology. Recent research results highlight the positive effects of PSO consumption from MCI patients, promoting cognition and memory improvement and an enhancement in processing and executive functions [32,33]. Consumption of pomegranate juice (PJ) by mouse AD models manifested reductions in Aβ deposition, fibrillar Aβ deposition, and hippocampal soluble Aβ_42_ compared to controls [34]. In addition, a diet supplemented with pomegranate extract attenuates AD pathology in a 12-month-old transgenic mouse model compared to the control [35]. Moreover, pomegranate leaf extract reversed the adverse effects of human Aβ_42_-induced neurodegeneration in transgenic Drosophila melanogaster [36]. However, to the moment, no clinical study has evaluated the effect of PSO consumption on crucial AD molecular biomarkers. 

Considering this supporting evidence, the present study aimed to investigate the potential therapeutic benefits of pomegranate seed oil (PSO) consumption on neuronal cell lines and patients diagnosed with MCI. Firstly, the study sought to evaluate the anti-inflammatory and antioxidant effects of PSO in murine neuroblastoma N2a cells treated with lipopolysaccharides (LPS), to mimic an AD-like inflammatory response. For this, crucial inflammation-related biomarkers (IL-1β, TNF-α, inducible NO synthase (iNOS)) and the redox marker SOD1 were determined in cell lysates, in the presence or absence of PSO. Additionally, the study aimed to assess changes in key AD-related biomarkers, including APP, tau protein, Aβ_42_, and tau phosphorylated at threonine-181 (p-tau181). Finally, we evaluated the impact of supervised PSO consumption for 12 months on Aβ_40_, Aβ_42_, p-tau181, and TNF-a, in the blood serum of MCI patients. The data reinforces the notion that inflammatory appeasement correlates with benefits in AD-related pathology.

## 2. Materials and Methods

### 2.1. Pomegranate Seed Oil

The PSO was extracted from organic pomegranate seeds by the cold extraction method and was kindly provided to us by Rodi Hellas (Pom Star), in Pella, Greece (GR-BIO-02). The extract has been analyzed by the Agrolab RDS, and it is accredited with all the necessary certifications. The chemical and physical properties and the fatty acid composition of the product have been reported by Chatzikostopoulos and Tsolaki (2022) [32]. PSO stock dilutions used in the in vitro studies were prepared in sterile DMSO (100 mg/mL).

### 2.2. Cell Culture

The murine neuroblastoma cell line Neuro-2a (N2a) (ATCC: CCL-131™) was chosen as a neuronal cell model. N2a cells were kindly provided by Prof. Gaetano Donofrio from the Department of Medical-Veterinary Science of the University of Parma, Italy. Cells were routinely cultured in TC-treated Cell Culture Flasks with Filter Cap (25 cm^2^ #430639, or 75 cm^2^ #430641, Corning, Corning NY, USA), with standard DMEM medium (4.5 g/L glucose, with L-glutamine, #LM-D1111/500, Biosera, Cholet, France), supplemented with 10% FBS (#10270106, Gibco, UK), 2 mM stable glutamine (#P04-82100, Pan-Biotech, Aidenbach, Germany), 100 I.U./mL penicillin and 100 μg/mL streptomycin (#XC-A4122/100, Biosera, Cholet, France). Cultures were kept at 37 °C, 5% CO_2_, in a humidified incubator. Growth medium was replenished every 2–3 days, and cultures were split 2 times per week. Cell stocks were kept at −80 °C, in a freezing medium containing 50% FBS, 10% DMSO, and 40% growth medium. All processes were performed in sterile conditions, in a laminar-flow hood. All employed reagents and solutions were endotoxin-free and sterile-filtered before use or provided sterile by the manufacturer.

#### 2.2.1. MTT Viability Assay

To assess the possible cytotoxic effect of the employed PSO, 10^4^ N2a cells were seeded in 96-well TC plates (#3596, Corning, Corning, NY, USA) for 16 h. The following day, medium was removed, cells were washed with Hank’s balanced salt solution (HBSS, #LM-S2040/500, Biosera, Cholet, France), and medium containing 0.5% FBS was added to induce starvation for 2 h. After that, the medium was removed, cells were washed, and PSO diluted in starvation medium (0.1 up to 100 μg/mL) was added to the wells. Following a 24-h incubation, the medium was removed, cells were washed with HBSS, and 0.5 mg/mL (final concentration) of 3-(4,5-dimethylthiazol-2-yl)-2,5-diphenyltetrazolium bromide (MTT) (#A2231, AppliChem, Darmstadt, Germamy) was added to each well. Cells were incubated for 3 h in the incubator, and the formed formazan crystals were washed once with Dulbecco’s phosphate-buffered solution (DPBS, #LM-S2041/500 Biosera, Cholet, France) and then dissolved with a solution containing 90% (*v*/*v*) isopropanol, 10% (*v*/*v*) Triton X-100 and 1 drop of 12 N HCl. Absorbance of the solution of the dissolved crystals was determined spectrophotometrically at 570 nm, and the viability of the cells was determined by setting the absorbance of the untreated control cultures as 100%.

Viability = A_Sample_ × 100/A_Control_ %.

The isopropanol—Triton X-100—HCl solution was used as a blank.

#### 2.2.2. Assessment of the Antioxidant Potential of PSO with MDA Determination

To assess whether PSO can protect cells from oxidative stress, we employed a common oxidative stress inducer, H_2_O_2_, followed by the subsequent determination of MDA titers. To do so, 2 × 10^5^ cells were seeded on 12-well TC plates (#665180, Greiner Bio-one, Kremsmünster, Austria) and grown for 16 h. Then, cells were washed with HBSS and incubated for 2 h in standard medium (control) or the medium supplemented with 0.2, 10, or 25 μg/mL of PSO. Then, cells were washed once more and incubated for 2 h in starvation medium (0.5% FBS) with the addition of the respective PSO concentrations. Finally, 100 μM H_2_O_2_ was added to the cells for 2 h, to induce oxidative stress. Cultures without the addition of H_2_O_2_ served as untreated cultures, in all cases. Medium was removed, cells were washed with DPBS, and lysed with a solution containing 0.1% (*v*/*v*) Triton X-100 in DPBS. Lysates were centrifuged (14,000 rpm, 15 min) to remove cell debris and 1 volume of 10% trichloroacetic acid (TCA) was added to the supernatants, followed by 1 volume of 0.67% (*w*/*v*) thiobarbituric acid (TBA, #A4670, AppliChem, Darmstadt, Germamy), dissolved in 0.05 M NaOH. Mixtures were then incubated for 1 h at 95 °C, centrifuged (14,000 rpm, 15 min), and the absorbance of the supernatants at 530 nm was determined. The concentration of MDA was determined by constructing a standard curve of MDA (#63287, Sigma Aldrich Chemie GmbH, Steinheim, Germany), which was treated in the same manner as the samples (Supplementary Figure S1).

#### 2.2.3. Treatment of LPS-Induced N2a Cells with PSO

To produce lysates for further analysis of crucial AD-related proteins, 2 × 10^5^ N2a cells were seeded on 6-well plates (#657160, Greiner Bio-one, Frickenhausen, Germany) and cultured under standard conditions overnight. The following day, cells were exposed to 0.2, 10, or 25 μg/mL of PSO for 2 h, then starved for 2 h in the presence of fresh PSO and finally, inflammation was induced with 1 μg/mL of LPS (#LPS25, Millipore, Burlington, MA, USA), for 24 h. Cultures without LPS and/or PSO were also used as adequate controls. After treatment, media were discarded, cells were washed with PBS twice and lysed in a buffer containing 50 mM Tris-HCl (pH 7.4), 150 mM NaCl, 1 mM EDTA, 1% (*v*/*v*) Triton X-100, 1% (*w*/*v*) deoxycholate sodium, 0.1% (*w*/*v*) SDS and 0.5% (*v*/*v*) protease inhibitor (#539134, Millipore, USA), at 4 °C, for 30 min. Lysates were centrifuged (14,000 rpm, 15 min) to remove debris and stored at −20 °C until analysis. Protein content of the lysates was determined with bicinchoninic acid (BCA) assay kit (#701780, Cayman Chemical, Ann Arbor, MI, USA).

#### 2.2.4. Western Blotting Analysis

A total of 15 μg of proteins from each cell lysate were separated by denaturing on 10% or 12% (*w*/*v*) SDS-polyacrylamide gel electrophoresis (SDS-PAGE), employing a Labnet ENDURO VE10 device. For the electrophoresis run, a buffer containing 0.303% (*w*/*v*) Tris-base, 1.44% (*w*/*v*) glycine, and 0.1% (*w*/*v*) SDS (pH 8.3) was used. Separated proteins were transferred on a 0.45 µM nitrocellulose membrane (#71208.01, Serva Electrophoresis GmbH, Heidelberg, Germany), at 4 °C, with a constant current of 350 mA (maximum voltage 100 V) for 1 h [transfer buffer: 0.303% (*w*/*v*) Tris-base, 1.44% (*w*/*v*) glycine, 0.02% (*w*/*v*) SDS, 10% (*v*/*v*) methanol, pH 8.3].

The membranes were blocked at room temperature, with gentle shaking for 1 h with 5% (*w*/*v*) skimmed milk in Tris-buffered saline (TBS, 50 mM Tris-HCl pH 7.6, 150 mM NaCl), containing 0.5% (*v*/*v*) Tween-20 (TBS-T). For the detection of APP, Aβ_42_, tau, p-tau181, iNOS, IL-1β, TNF-α and SOD1, the following primary antibodies were employed: anti-APP mouse monoclonal antibody (#60342-1-Ig, Proteintech, Manchester, UK), anti-Aβ_42_ rabbit monoclonal antibody (#14974, Cell Signaling Technology, Danvers, MA, USA), anti-rabbit tau monoclonal antibody (#46687, Cell Signaling Technology, Danvers, MA, USA), anti-p-tau181 rabbit monoclonal antibody (#12885, Cell Signaling Technology, Danvers, MA, USA), anti-iNOS rabbit polyclonal antibody (#18985-1-AP, Proteintech, Manchester, UK), anti-IL-1β rabbit monoclonal antibody (#L0328Y, Cusabio, Houston, TX, USA), anti-TNF-α mouse monoclonal antibody (#sc-52746, Santa Cruz Biotechnology, Heidelberg, Germany), and an anti-SOD1 rabbit polyclonal antibody (#10269-1-AP, Proteintech, Manchester, UK). Two horseradish peroxidase (HRP)-conjugated antibodies were used as secondary antibodies: the anti-mouse IgG binding protein m-IgGκ BP-B (#sc-516142, Santa Cruz Biotechnology, Heidelberg, Germany) and the goat anti-rabbit IgG (#SA00002-2, Proteintech, Manchester, UK). In addition, an anti-β-actin monoclonal mouse antibody (#sc-47778, Santa Cruz Biotechnology, Heidelberg, Germany) was employed as internal control to confirm equal protein loading, and to verify the results of Ponceau S Staining (0.1% *w*/*v* in 5% *v*/*v* ethanoic acid) of the transferred proteins on the nitrocellulose membrane (see Supplementary Figure S2). Ponceau staining was applied every time, before immunoblotting, to verify efficient transfer and equal protein loading, as previously suggested [37]. The antibodies were diluted in 5% (*w*/*v*) bovine serum albumin in TBS-T. More details on antibody dilutions are provided in the Supplementary Table S1.

Membranes were incubated with the antibodies for 1.5 h at room temperature. Incubations performed with primary or secondary antibodies was followed by three 10-min washes with TBS-T. The specific protein bands were visualized at ChemiDoc imaging system (Biorad), after immersing the membranes for 1 min in FastGene Western ECL reagent (#FG-CH01, Nippon Genetics, Tokyo, Japan). All markers detected on a single membrane (see Supplementary File) were exposed with the same settings and time, and all had their representative loading control. The density of the bands was semi-quantified with ImageJ 1.49 software (National Institutes of Health—NIH, Bethesda, MD, USA) [38].

#### 2.2.5. Statistical Analysis

GraphPad Prism 8 (GraphPad Software Inc.) was employed for the statistical analysis and the design of all graphs. The graphs represent arbitrary density units of each protein target, divided by the corresponding value of β-actin (equal loading control), and finally were normalized to present the value of the control sample as 1. Bar heights correspond to mean values ± standard deviations (SD), derived from three independent experiments (Supplementary Section S1.2 for replicate membranes). To predict possible statistical significance among the different mean values obtained from Western blot analyses, ordinary one-way ANOVA, without correction for multiple comparisons (Uncorrected Fisher’s LSD test), was employed after verifying Gaussian distribution by normality test of Shapiro-Wilk. Asterisks in graphs represent statistically significant differences between the control and LPS-treated sample, or the samples treated only with PSO. Hashtags in graphs represent statistically significant differences between LPS-treated samples and samples treated with both LPS and PSO.

Two-way ANOVA (Uncorrected Fisher’s LSD test) was employed for statistical analyses of the MDA test. Asterisks in graphs represent statistically significant differences between the control and H_2_O_2_-treated samples. Hashtags in graphs represent statistically significant differences between H_2_O_2_-treated samples and samples treated with both H_2_O_2_ and PSO, and dollars represent statistically significant differences between the control sample and samples treated only with PSO.

The statistical significance was set at *p* ˂ 0.05 in all cases. Statistical significance notations: * *p* < 0.05, ** *p* < 0.01, *** *p* < 0.001, **** *p* < 0.0001; # *p* < 0.05, ## *p* < 0.01, ### *p* < 0.001, #### *p* < 0.0001.

### 2.3. Evaluation of the Effect of PSO Consumption on MCI Patients

#### 2.3.1. Subjects

The participants originated from a Mediterranean area (Thessaloniki region, northern Greece). All subjects were white, community-dwelling individuals, and native Greek speakers. Participants were literate and healthy (were not suffering from any debilitating disease) as ascertained from their medical history and physical and neurological examination. All participants were recruited from the Greek Association of Alzheimer’s Disease and Related Disorders’ Day Care Centers “Saint John” and “Saint Helen” (Alzheimer Hellas, DCCSJSH). All the participants followed the same processes, which included clinical examination, laboratory/imaging procedures, and neuropsychological assessment. The laboratory results were evaluated by the neurologist of the Greek Association of Alzheimer’s Disease and Related Disorders (GAADRD). The exclusion criteria were hearing deficits, visual deficits, no comprehension of the Greek language, drug treatment, use of antipsychotics, and a score on the Geriatric Depression Scale higher than 6 [32].

The demographic characteristics of all participants are presented in Table 1, as mean values ± SD.

#### 2.3.2. Study Design

The study was approved by the Scientific and Ethics Committee of the GAADRD (Scientific Committee Approved Meeting Number: 71/07-10-2021), and followed the new General Data Protection Regulation (EU) 2016/679 of the European Parliament and of the Council of 27 April 2016 on the protection of natural persons with regards to the processing of personal data and on the free movement of such data, as well as the principles outlined in the Helsinki Declaration for experimentation with human subjects [39].

All participants were informed and gave their consent for the use of demographic data. All individuals were subjected to neuropsychological assessment and blood serum drawing before the start of the study (Baseline) and at the end of the study, after 12 months (12 mo.).

MCI patients were randomly divided into 4 groups with SPSS v.27 statistical software: Control Baseline (n = 23) and Control 12 mo. (n = 23), PSO Baseline (n = 31), and PSO 12 mo. (n = 31). At the PSO group, patients consumed 5 drops of PSO daily for 12 months and followed the Mediterranean Diet (MeDi). Compliance with MeDi and PSO consumption was observed through a questionnaire and a dairy calendar. The demographic data of all participants is summarized in Table 1. The PSO that was administered to the patients was donated by the Pom Star Rodi Hellas company. The detailed physical and chemical content of PSO is demonstrated in Chatzikostopoulos (2022) [32].

#### 2.3.3. Processing of Blood Serum Samples

Whole blood samples were collected from subjects in the morning hours, before the onset of the study (Baseline), and after its completion (After one year of study). Blood samples were allowed to clot for 30 min at room temperature in appropriate serum separator tubes. Following centrifugation for 20 min at 1000× *g*, sera were collected, aliquoted, and stored at −80 °C until analysis. Care has been taken to avoid multiple freeze-thaw cycles. Necessary dilutions of the sera, before the analyses were performed with commercial dilution buffer provided with the kits described below, just before the analyses.

#### 2.3.4. Analysis of Biomarkers Levels by ELISA

Biomarkers in the sera of the participants were detected by commercial sandwich, HRP-conjugated ELISA kits provided by Assay Genie (Dublin, Ireland), as follows: Human Amyloid Beta 40/AB 1-40 ELISA Kit (#HUFI02244), Human Amyloid Beta 42/AB 1-42 ELISA Kit (#HUFI02245), Human Phospho Tau (P181) ELISA Kit (#HUFI03189), Human TNF alpha ELISA Kit (#HUFI00262).

All analyses were performed in duplicates, in blind, according to the manufacturer’s instructions by two independent researchers. Quantification of biomarkers in blood serum samples was performed after the construction of standard curves, employing protein standards included in the kits. Double-distilled water (d.d. H_2_O) was used in all cases when needed.

#### 2.3.5. Statistical Analysis for PSO Effect on MCI Patients

Statistical analysis and graphs were conducted with GraphPad Prism 8 (GraphPad Software Inc.). All bars depict mean values ± SD.

Participants’ demographic and clinical characteristics were evaluated with standard unpaired *t*-tests. For MMSE, a nonparametric *t*-test was conducted to compare differences between the same group and the Mann-Whitney test for comparison, between the Controls versus the PSO group. As for gender, Chi-Squared analysis was used.

The statistical tests exploited in this study were chosen after performing the analyses for normal (Gaussian) distribution and homogeneity of the data, performed with the tests of Anderson-Darling and Bartlett, respectively. To examine possible discrimination for biomarkers’ levels between baseline and after the performance of the interventions, a paired and parametric *t*-test was employed for the TNF-α PSO group, for Aβ_42_, and Aβ_42_/Aβ_40_. A paired and nonparametric *t*-test was used when the parameters did not follow the Gaussian Normality between the same groups (TNF-α Control, Aβ_40_, p-tau181, ratio of p-tau181/Aβ_42_). Moreover, an unpaired *t*-test was performed to examine discrimination between Control 12 mo. and PSO 12 mo. Asterisks represent statistically significant differences between baseline measurements at 12-month, and the hashtags represent differences between the biomarker levels of the control group and the PSO group at the follow-up. Correlation analysis with Spearman’s test has been used to determine the relationships among analyzed variables.

The statistical significance was set at *p* ˂ 0.05 in all cases. Statistical significance notations: * *p* < 0.05, ** *p* < 0.01, *** *p* < 0.001, **** *p* < 0.0001; # *p* < 0.05, ## *p* < 0.01, ### *p* < 0.001, #### *p* < 0.0001.

## 3. Results

### 3.1. The Βeneficical Effect οf PSO on LPS-Stimulated N2a Murine Neuroblastoma Cells

#### 3.1.1. Tolerability of N2a Cells to PSO

A viability assay was performed to assess the tolerability of the N2a cells to PSO. The results of the study are depicted in Supplementary Figure S3. In the employed PSO concentrations (up to 100 μg/mL), no statistically significant reduction in N2a viability was demonstrated. Thus, it was regarded that the use of PSO concentrations up to 25 μg/mL would be safe.

#### 3.1.2. PSO Treatment Attenuated the Overexpression of Amyloid Triggered by LPS

**Figure 1:**
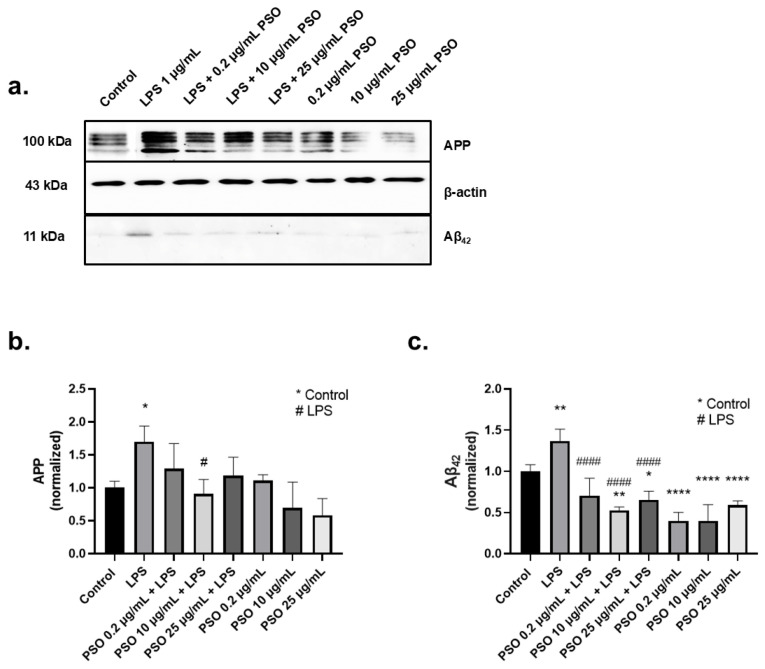
Effects of PSO on APP and Aβ_42_ levels in N2a cells challenged with LPS. (**a**) Representative Western blot images showing the levels of APP and Aβ_42_ in N2a cells treated with 1 μg/mL LPS in the absence or presence of PSO at varying concentrations (0.2, 10, and 25 μg/mL) (see Supplementary Material Section S1.1). (**b**) Quantification of APP levels normalized to β-actin. (**c**) Aβ_42_ levels normalized to β-actin. Data are presented as mean ± SD from three independent experiments. Statistical significance: * *p* < 0.05; ** *p* < 0.01; **** *p* < 0.0001, with asterisk (*) indicating the comparison versus control group; # *p* < 0.05; #### *p* < 0.0001, with (#) indicating the comparison with the LPS-treated group.

Bacterial LPS affect key physiopathological aspects of AD, such as Aβ regulation, tau protein abnormalities, neuroinflammation, and neurodegeneration [40]. Consequently, are often employed to stimulate neuroinflammation and mimic AD-like characteristics [41]. For that reason, N2a cells were pre-treated with PSO (0.2 μg/mL, 10 μg/mL, and 25 μg/mL) for 4 h, and subsequently they were exposed to 1 μg/mL of LPS for 24 h. LPS increased by 1.7-fold the levels of APP, compared to the control (*p* = 0.0275; Figure 1a,b) and the levels of Aβ_42_ by 1.4-fold compared to the control (*p* = 0.0034; Figure 1a,c). The addition of 10 μg/mL of PSO in N2a cells reduced the LPS-induced amyloid exacerbation (−80%, *p* = 0.0119 for APP; −88%, *p* = 0.0004 for Aβ_42_). Treatments with PSO in the absence of LPS had no statistically significant effect on APP. Besides, Aβ_42_ levels were reduced with all PSO treatments compared to control (*p* < 0.0001) (Figure 1b,c).

**Figure 2:**
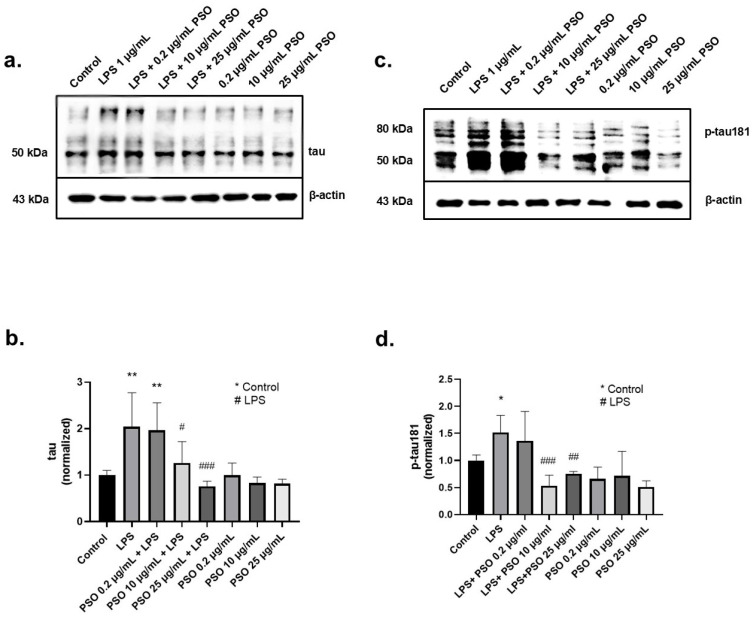
The effects of PSO on LPS-induced tau and phosphorylated tau (p-tau181) levels in N2a cells. (**a**,**c**) Representative Western blot images showing the expression levels of tau and p-tau181 in N2a cell (see Supplementary Material Section S1.1). (**b**) Quantification of tau protein levels normalized to β-actin. (**d**) Quantification of p-tau181 levels normalized to β-actin. Statistical significance: * *p* < 0.05; ** *p* < 0.01, with asterisk (*) indicating the comparison versus control group; # *p* < 0.05, ## *p* < 0.01, ### *p* < 0.001, with (#) indicating the comparison with the LPS-treated group. Data are presented as the mean ± SD.

AD is closely related to the overexpression of tau and p-tau181. The tau protein levels were determined after 24 h of exposure to PSO, in the presence or absence of LPS. As shown in Figure 2a, LPS induced an increase of 2.0-folds in tau levels (*p* = 0.0049; Figure 2b), compared to baseline levels. The effect of LPS was mitigated by PSO in a concentration-dependent manner, with 25 μg/mL being the most effective concentration reducing p-tau181 levels to −124% (*p* = 0.0010).

Exposure of N2a cells to LPS for 24 h resulted in a 1.5-fold increase of p-tau181 levels compared to control (*p* = 0.0496; Figure 2c). Pre-treatment of LPS-induced N2a cells with 10 μg/mL of PSO effectively mitigated this increase at baseline levels (*p* = 0.0009; Figure 2c). PSO alone had no impact on tau and p-tau181 levels. These results imply that the PSO acts as a modulator of the tau physiology under inflammatory stress conditions (LPS exposure).

#### 3.1.3. PSO Mediates a Potent Anti-Inflammatory Effect on N2a Cells

**Figure 3:**
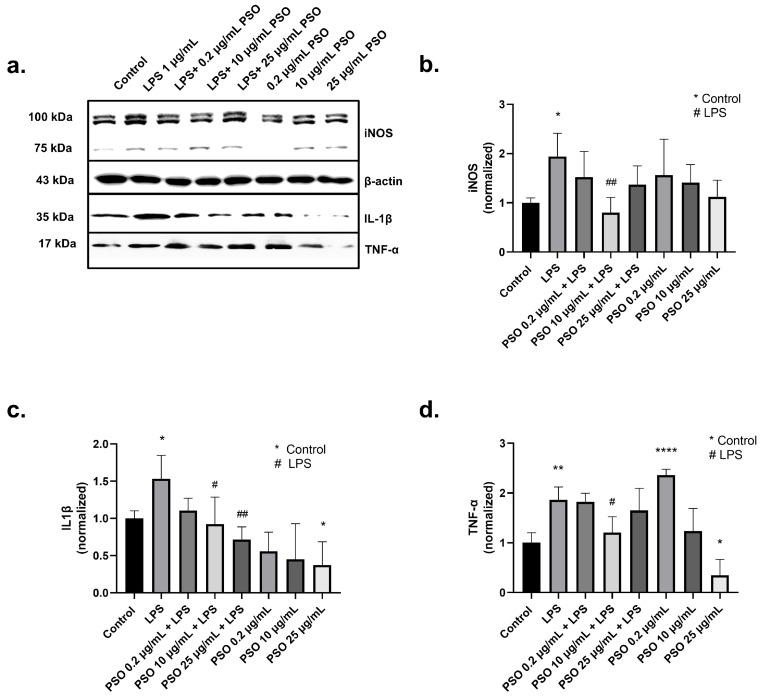
The effects of PSO on LPS-induced expression of inflammatory and oxidative stress markers in N2a cells treated with PSO. (**a**) Representative Western blot images showing the expression levels of inducible nitric oxide synthase (iNOS), interleukin-1β (IL-1β), and tumor necrosis factor-alpha (TNF-α). Groups include Control, LPS (1 µg/ mL), LPS co-treatment with different concentrations of PSO (0.2, 10, and 25 µg/mL), and PSO (0.2, 10, and 25 µg/mL) (see Supplementary Material Section S1.1). (**b**) Quantification of total iNOS protein levels normalized to β-actin. (**c**) Quantification of IL-1β protein levels normalized to β-actin. (**d**) Quantification of TNF-α protein levels normalized to β-actin. Statistical significance: * *p* < 0.05; ** *p* < 0.01; **** *p* < 0.0001, with asterisk (*) indicating the comparison versus control group; # *p* < 0.05, ## *p* < 0.01, with (#) indicating the comparison with the LPS-treated group. Data are presented as the mean ± SD.

iNOS is regarded as one of the key molecular mediators of LPS-related inflammation and iNOS levels are elevated during inflammation [42]. Treatment of N2a cells with 1 μg/mL LPS caused a statistically significant increase by 1.94-fold in the expression of total iNOS compared to control samples (*p* = 0.0180; Figure 3a,b). However, in the presence of PSO, iNOS levels were reduced in comparison to LPS-treated cells (Figure 3b), with the most intense reduction observed by 10 μg/mL of PSO (−114%, *p* = 0.0056; Figure 3a,b).

An interplay between IL-1β overexpression and AD onset has been demonstrated, while Aβ deposits strongly correlate with IL-1β levels [43,44]. In our system, treatment of N2a cells with LPS increased IL-1β levels 1.53-fold (Figure 3a,c), compared to control samples (*p* = 0.0401). Similarly, TNF-α levels were increased by 1.86-fold after exposure to LPS (*p* = 0.0034; Figure 3a,d). PSO at 10 μg/mL significantly reduced IL1β levels in LPS-treated N2a cells (−62%, *p* = 0.021; Figure 3a,c) concomitantly with a reduction in TNF-α levels (−66%, *p* = 0.0188; Figure 3a,d). Therefore, the induction of TNF-α levels by 0.2 μg/mL PSO can be attributed to an adaptive cellular response. Collectively, these results demonstrate the anti-inflammatory potential of PSO under stress conditions, as those caused by LPS.

#### 3.1.4. PSO Exerts an Antioxidant Activity in N2a Cells

**Figure 4:**
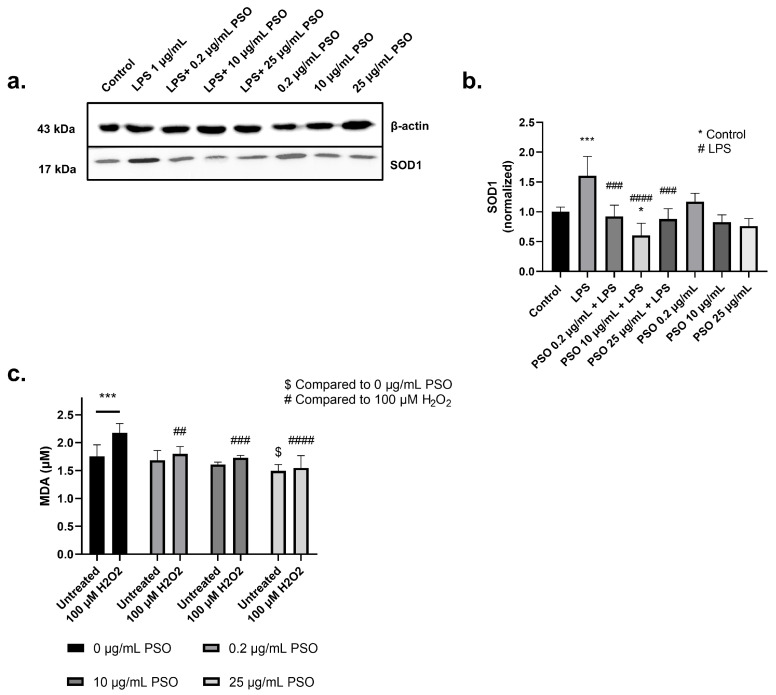
(**a**) Representative Western blot images showed the expression levels of superoxide dismutase 1 (SOD1) across different experimental conditions (see Supplementary Material Section S1.1). (**b**) Quantification of SOD1 protein levels normalized to β-actin. (**c**) Measurement of malondialdehyde (MDA) levels under oxidative stress conditions induced by 100 µM H_2_O_2_. Untreated and H_2_O_2_-treated groups were analyzed in the presence of different PSO concentrations (0, 0.2, 10, and 25 µg/mL). Statistical significance: * *p* < 0.05; *** *p* < 0.001, with asterisk (*) indicating the comparison versus control group; ## *p* < 0.01, ### *p* < 0.001, #### *p* < 0.0001, with (#) indicating the comparison versus H_2_O_2_; $ *p* < 0.05, with ($) indicating the comparison versus 0 µg/mL PSO. Data are presented as the mean ± SD.

To evaluate the antioxidant capacity of PSO we determined the levels of SOD1 in PSO-treated cells. As presented in Figure 4a,b, LPS treatment significantly increased the levels of SOD1 in N2a cells (1.60-fold, *p* = 0.0010), demonstrating the induction of oxidative stress. On the other hand, the presence of PSO reinforced the antioxidant defense of the N2a cells against LPS, as indicated by the reduction in the SOD1 levels with 10 μg/mL of PSO to levels even below those of the control cells (−100%, *p* = 0.0183; Figure 4a,b). PSO did not affect SOD1 levels in the absence of LPS.

To further explore the antioxidant activity of PSO, the oxidative status of cells challenged with H_2_O_2_ in the presence or absence of PSO was also evaluated via the determination of the peroxidation marker MDA (Figure 4c). As anticipated, MDA levels increased by 23.8% in cells challenged with H_2_O_2_ (*p* = 0.0006), while PSO at all employed dosages (0.2, 10 and 25 μg/mL) inhibited this effect (−13.8%, *p* = 0.0010; −20.3%, *p* = 0.0003; −28.8%, *p* < 0.0001; respectively). Surprisingly, PSO at 25 μg/mL also lowered the basal MDA levels, in comparison to the control culture (−14.8%, *p* = 0.0133). These results further demonstrate the antioxidant capacity of PSO.

### 3.2. The Impact of Pomegranate Seed Oil on MCI Patients: A Clinical Evaluation

#### 3.2.1. Statistical Analysis of Subjects’ Demographics

Participants’ demographics and the *p*-values of the statistical analyses are summarized in Table 1, while the values displayed represent mean values ± SD. The comparison of the MMSE scores of the cohorts is also presented in Supplementary Figure S4. No significant differences were observed for the studied demographics, except for the MMSE score. In detail, MCI patients who continuously consumed PSO for one year performed better in the MMSE test (28.45 ± 1.87) compared to the PSO baseline (27.65 ± 1.38; *p* = 0.0046). In addition, the comparison between the group that consumed PSO for 12 mo. and the Control MCI group after 12 mo. suggests that the MMSE score of MCI patients reached significantly higher levels after intervention (*p* = 0.0015).

#### 3.2.2. Consumption of PSO for 12 Months Improved the Ratio of Aβ_42_/Aβ_40_

**Figure 5:**
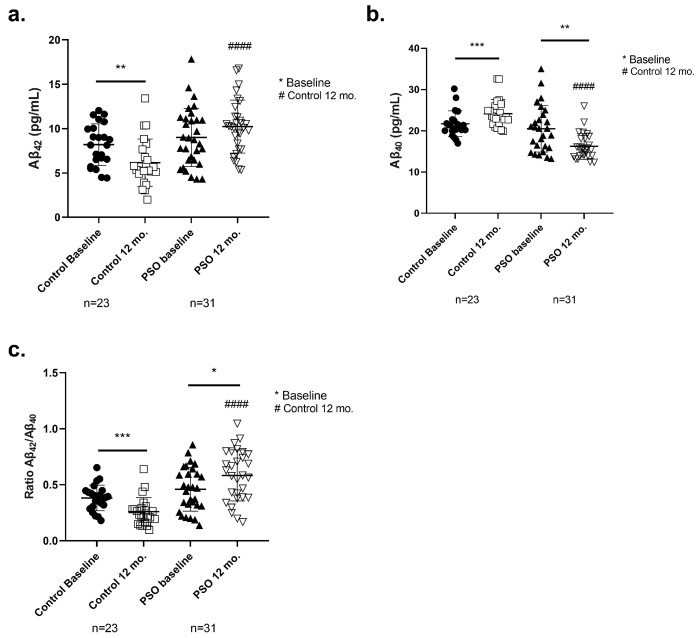
Levels of (**a**) amyloid peptide 1-42 (Aβ_42_), (**b**) amyloid peptide 1-40 (Aβ_40_) determined by ELISA, and (**c**) the ratio Aβ_42_/Aβ_40_ (Aβ_42_/Aβ_40_) in blood sera from patients with MCI who did not receive PSO (Controls) (n = 31) at the beginning and at the end of the trial compared to MCI patients who received no pharmacological therapy with PSO (n = 31). Results are provided with individual values scatter plots as mean values ± standard deviation (SD). (**c**) The calculation of the ratio Aβ_42_/Aβ_40_. All samples were analyzed at least in duplicate. Statistical analyses were performed with GraphPad Prism 8.0 statistical software. A *t*-test was used to examine possible differences between groups. * *p* < 0.05; ** *p* < 0.01, *** *p* < 0.001, with (*) indicating differences between 12 mo. and baseline levels. #### *p* < 0.0001, with (#) indicating differences between follow-up (12 mo.) levels of the Control and PSO groups.

Peptides Aβ_40_ and Aβ_42_, the main beta-amyloid species, have been analyzed in the serum of the participants of the present study. In addition, the ratio of Aβ_42_ to Aβ_40_ (Aβ_42_/Aβ_40_) was also calculated to estimate the amyloid pathology of the patients. The mean levels (±SD) of amyloid peptides and their ratio for every studied group, at the baseline and 12 months later, are provided in Table 2, and the distribution of those is depicted in Figure 5.

Higher levels of serum Aβ_42_ correlate with greater clearance of the amyloid burden in the brain [45,46]. The respective concentrations of the Aβ_42_ levels of each group and the *p*-values are summarized in detail in Table 2. The PSO group, at the follow-up, displayed higher levels of Aβ_42_ (10.22 ± 2.984 pg/mL), compared to the Control 12-month group, which showed significantly decreased levels of Aβ_42_ at 12 mo. (6.157 ± 2.671 pg/mL, *p* < 0.0001). In addition, patients who did not follow the PSO presented worse amyloid profiles, as Aβ_42_ levels decreased compared to their respective baseline (*p* = 0.0025).

Within the 12 months of the study, Aβ_40_ levels were increased in MCI patients of the control group (24.15 ± 3.385 pg/mL) compared to the beginning of the study (21.70 ± 3.123 pg/mL, *p* = 0.0002). This effect possibly correlates with increased proteolysis and/or overexpression of APP linked to prodromal AD stages. PSO intervention for 12 months, significantly reduced the levels of Aβ_40_ (16.22 ± 3.144 pg/mL) in comparison to the MCI control group (20.48 ± 5.628 pg/mL; *p* = 0.0014). Moreover, significantly different levels were observed between the PSO intervention group at 12 mo., compared to the MCI patients not following any intervention (*p* < 0.0001) (Figure 5b).

Increased ratio of Aβ_42_/Aβ_40_ is considered a defining factor for the improvement of amyloid [47]. The Aβ_42_/Aβ_40_ ratio was significantly decreased in the MCI patients of the control group after the completion of the study compared to baseline levels (−32%, *p* < 0.0001), indicating the inability to clear toxic Aβ_42_ species from the brain. On the other hand, patients who consumed PSO for 12 mo. presented a significantly increased ratio compared to the respective baseline (+27%, *p* = 0.0146) and the MCI Control group (+225%, *p* < 0.0001).

#### 3.2.3. The Consumption of PSO for 12 Months Reduced p-tau181 Levels in MCI Patients

**Figure 6:**
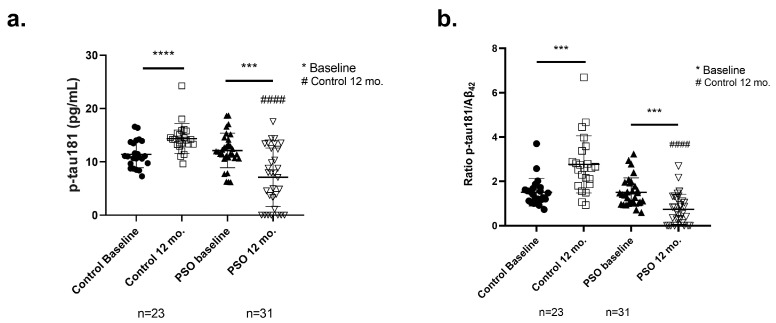
Levels of (**a**) tau protein phosphorylated at Threonine-181 (p-tau181) and (**b**) of the ratio p-tau181/Aβ_42_ as measured from the implemented ELISA on patients with MCI who did not receive PSO (Controls) (n = 31) at the beginning and at the end of the trial compared to MCI patients who received non-pharmacological therapy with PSO (n = 31) at the beginning and at the end of the trial as well. Results are provided with individual values scatter plots, which show mean values ± standard deviation (SD). All samples were analyzed at least in duplicate. Statistical analyses were performed with GraphPad Prism 8.0 statistical software. A *t*-test was used to examine possible differences between groups. *** *p* < 0.001, **** *p* < 0.0001, with (*) indicating differences between 12 mo. and baseline levels. #### *p* < 0.0001, with (#) indicating differences between follow-up (12 mo.) levels of the Control and PSO groups.

P-tau181 levels in blood serum have been identified as a diagnostic marker for AD [48]. For that reason, we examined the effect of PSO consumption on the levels of p-tau-181 in the serum of all participants (Table 2 and Figure 6a). Notably, decreased levels of p-tau-181 were observed at the end of the trial in the serum of MCI participants who received PSO, compared to the baseline levels (7.106 ± 5.485 pg/mL versus 12.13 ± 3.247 pg/mL; *p* < 0.0001). On the other hand, MCI patients of the control group that did not consume PSO, presented significantly increased p-tau181 serum titers (14.36 ± 2.862 pg/mL), when compared with both their respective baseline (*p* < 0.0001) and patients that received PSO for 12 mo. (*p* = 0.0001).

The ratio p-tau181/Aβ_42_ reflects the general AD pathology as it strongly correlates with protein aggregates and disease progression [49,50,51]. By calculating the ratio of p-tau181/Aβ_42_ (Table 2 and Figure 6b), we observed, after the end of the study, a significantly lower ratio in patients who followed the PSO intervention, compared to those who did not receive PSO (−73%, *p* < 0.0001). In addition, a significant reduction in the ratio levels is documented in patients who stick to PSO supplementation, compared to their respective baseline levels (−51%, *p* = 0.0004). On the other hand, patients who did not follow any intervention presented an increase of 83% in the ratio (*p* = 0.0004). These outputs may reflect the ameliorative effect of PSO consumption in the progression.

#### 3.2.4. PSO Consumption Reduced TNF-α Levels in Sera of MCI Patients

**Figure 7:**
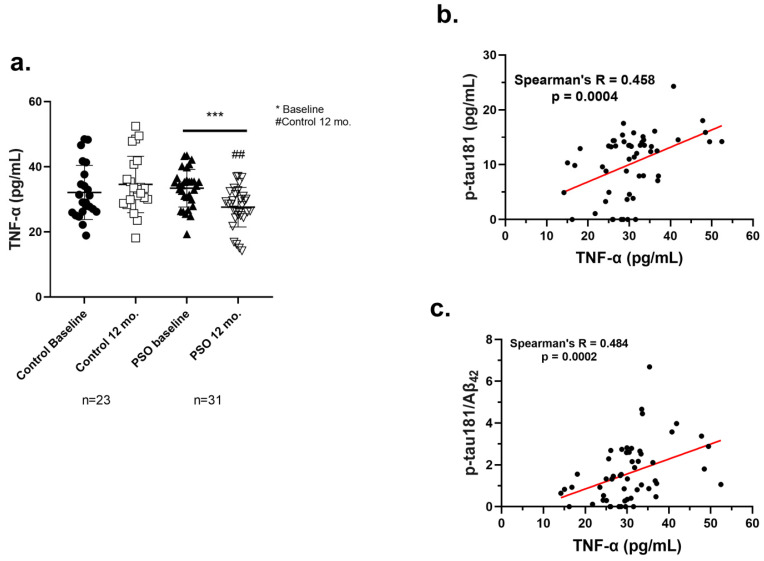
(**a**) Levels of TNF-α as measured from the implemented ELISA on patients with MCI who did not receive PSO (Controls) (n = 23) at the beginning and at the end of the trial compared to MCI patients who received no pharmacological therapy with PSO (n = 31). Results are provided with individual values scatter plots which mean values ± standard deviation (SD). All samples were analyzed at least in duplicates. Statistical analyses were performed with Graph Pad Prism 8.0 statistical software. *t*-test was used to examine possible differences between groups. * Differences between follow-up and baseline levels. # Differences between follow-up (12 mo.) levels of the Control group and the PSO group. *** *p* < 0.001, with (*) indicating differences between 12 mo. and baseline levels. ## *p* < 0.01, with (#) indicating differences between follow-up (12 mo.) levels of the Control and PSO groups. (**b**) Correlation analysis of the levels of p-tau181 against the levels of TNF-a in blood serum of MCI patients employed in the current study. (**c**) Correlation analysis of the ratio of p-tau181/Aβ_42_ against the levels of TNF-α in blood serum of MCI patients of PSO study. The correlations were evaluated using Spearman’s rank correlation coefficients (r) and their corresponding *p* values. Analyses were run and graphed separately. Statistical analyses were performed with Graph Pad Prism 8.0 statistical software.

TNF-α is an inflammatory biomarker promptly correlated with a higher risk of AD onset [52]. TNF-α levels in the sera of all participants were determined with the ELISA method. The mean levels ± SD of this biomarker in each group are provided in Table 2, and the distribution of those is depicted in Figure 7a.

A significant beneficial impact concerning the inflammation through TNF-α levels was observed in the PSO group at the end of the study compared to the baseline levels (27.57 ± 6.098 pg/mL, versus 33.41 ± 5.771 pg/mL; *p* = 0.0008). Conversely, the Control 12 mo. group exhibited elevated levels of TNF-α compared to PSO 12 mo. patients (*p* = 0.0010). These data imply that PSO could exert an anti-inflammatory effect, with a beneficial impact on an inflammation-related disease.

#### 3.2.5. Positive Correlation of TNF-α with AD-Related Biomarkers

To further demonstrate the anti-inflammatory potential of PSO consumption in MCI patients, a correlation analysis was performed between the levels of TNF-α and the other biomarkers, determined at the beginning of the study and 12 months later, by the end of it. The analysis demonstrated that the levels of TNF-α correlate in a positive, linear manner with the levels of p-tau181 (Figure 7b) and the crucial ratio p-tau181/Aβ_42_ (Figure 7c) (Spearman’s R = 0.458, *p* = 0.0004; 0.484, *p* = 0.0002, respectively). The outcome of this analysis implies that the mitigation of inflammatory responses by PSO consumption demonstrated by the reduction of TNF-α, may be reflected in an improved amyloid-tau pathology in MCI patients.

## 4. Discussion

AD is the most prominent cause of dementia in the elderly, affecting over 50 million individuals worldwide [1]. Though several therapeutic schemes were introduced worldwide, including acetylcholinesterase (AChE) inhibitors, like Donezepil [53], and anti-Aβ monoclonal antibodies, like Aducanumab and Lecanemab [54,55], they have limited effect in preventing progression of the disease and, in some cases, are life-threatening. Threferore, there is still a pressing need for the development of a preventive or therapeutic scheme that would pose no other risks for the health of the patients. Among various approaches, a holistic dietary regimen rich in fruits, vegetables, nuts, grains, legumes, and seeds has garnered increasing attention for promoting brain health and delaying cognitive aging [19,24].

Pomegranate, which is rich in polyphenols, has a longstanding history of use in traditional medicine, and has neuroprotective effects reducing risk factors of AD progression Its content in several bioactive compounds, mainly polyphenols has been associated with numerous therapeutic properties, including antiviral, bactericidal, anti-inflammatory, and antioxidant [56]. In addition, the antioxidant capacity of pomegranate juice and seed oil surpasses that of many other fruits and is supposedly able to mitigate oxidative neuronal damage [57,58,59,60]. Several animal studies, as well as a clinical trial involving middle-aged and older adults have indicated the potential of pomegranate products for memory-enhancing. However, to this day, there have been sparse clinical data for the therapeutic potential of pomegranate consumption in dementia [32,33,61,62]. Therefore, in the present study, we aimed to examine the prophylactic effect of PSO (a) in vitro, on LPS-challenged neuroblastoma cells and (b) in vivo, by providing dietary supplementation of PSO for 12 months to patients diagnosed with amnestic MCI.

Neuroinflammation is a central event in AD pathology. To date, several studies suggest that systemic infections and neuroinflammation may contribute to the development of AD, proposing that early-life or lifelong exposure to infectious agents may be associated with an increased risk of developing dementia [63,64]. The implication of LPS—an endotoxin produced by Gram-negative bacteria, has been documented for the induction of neurodegenerative and neuroinflammatory phenomena and is implied as a mean for modelling neurodegenerative diseases both in vitro and in vivo [65,66]. LPS can induce APP overexpression and amyloid aggregation [67,68,69], upregulation of BACE1, overexpression of tau [66], and tau hyperphosphorylation [68,70]. Thus, we employed LPS in the current in vitro study, to mimic the AD neuroinflammatory pathology.

Several in vitro studies have indicated the regulatory effect of pomegranate in the central AD hallmarks, namely Aβ and p-tau. Nano forms of the polyphenolic fraction of pomegranate seed can inhibit and disaggregate amyloid fibril formations [71]. In addition, pomegranate extract has been reported to inhibit AChE—a crucial pharmacological target in AD management, the β-site amyloid precursor protein cleaving enzyme 1 (BACE1), thus reducing Aβ_42_ production [72,73]. These features were later attributed to the polyphenol gallagic acid and castalagin—an ellagitannin [74]. Furthermore, gallagic acid and castalagin decrease Aβ peptide secretion from N2a cells that overexpress the human APP Swedish mutation (N2a/APP cells). In addition, when N2a/APP were challenged with Aβ_42_ in the presence of gallagic acid, a significant reduction in ROS production was verified [74]. Recent research has also provided positive feedback on the effect of pomegranate compounds punicalagin (PUC), ellagic acid (EA), as well as peel, and aril extracts on tau phospho-homeostasis of Aβ_42_-induced H4 human neuroglioma cells. While Aβ_42_ significantly increased the titers of p-tau181, co-incubation with pomegranate arils or peel extracts, and PUC significantly attenuated p181 phosphorylation, and the effect was more prominent with EA treatment [75]. These results agree with the observations of the current study, as reduced levels of both Aβ_42_ and p-tau181 are documented with PSO treatment, compared to cells treated only with LPS (Figure 1a,c and Figure 2c,d). An additional observation of this study is the ability of PSO to prevent the overexpression of APP and tau, which is induced by LPS. This effect may assist in limiting the production and subsequent aggregation of neurotoxic peptides.

The alleviating potential of pomegranate against neuroinflammatory events has been previously proven. In a recent study by Alami et al., pomegranate peel or aril extracts attenuated LPS-induced inflammation of U373-MG human astrocytes and THP-1 human macrophages, as indicated by reduced titers of inflammatory cytokine IL-1 and increased levels of anti-inflammatory cytokine IL-10. In addition, pomegranate treatment promoted the M2 phenotype of microglial cells—a state correlating with anti-inflammatory and healing attributes, rather than prolonged and toxic neuroinflammation [75]. The activation of toll-like receptor 4 (TLR4) and subsequent triggering of Nuclear factor kappa beta (NF-κΒ) and mitogen-associated protein kinase (MAPK) pathways are core events in LPS-induced inflammation, eventually resulting in excess production of inflammatory cytotoxic factors, such as IL-1β, IL-6, TNF-α, NO, ROS and iNOS [76]. Therapeutic efforts in murine RAW264.7 macrophages indicated that pomegranate peel extracts inhibit TLR4/NF-κΒ pathway [76], iNOS and cyclooxygenase-2 expression [76], allegedly due to inhibition of NF-κB and MAPKs (ERK, JNK and p38) by PUC and EA [76,77]. Furthermore, a PSO dosage of 25 μg/mL can inhibit LPS-stimulated NO production, TNF-α release and iNOS in stimulated BV-2 murine microglia [78]. The aforementioned data agree with the results of the present study, where PSO extract mitigated LPS-induced inflammation against N2a cells, as proven by reduced titers of IL-1β, TNF-α and iNOS (Figure 3). Interestingly, we verified a pro-inflammatory effect of single low-PSO dosage (0.2 μg/mL) on iNOS levels (though not statistically significant), as also a significant increase in TNF-α levels. This effect, however, was inverted when employing higher PSO doses. Similar dose-dependent responses (hormesis effect) have been reported for other polyphenolic compounds, suggesting that initial pro-inflammatory signaling might act as a precursor for adaptive cellular responses that enhance resilience to oxidative and inflammatory stress [79]. The rich polyphenolic content of PSO, including punicic acid and flavonoids, may be responsible for modulating inflammatory mediators differently depending on the dosage [80].

Oxidative stress is a defined entity in neurodegenerative brain, with pathological hallmarks (Aβ plaques, NFTs) and inflammation-exacerbated glial cells being key mediators in the process [81,82]. A significant number of research cases have documented the antioxidant potential of pomegranate. Extracts of pomegranate peels demonstrate DPPH radical scavenging activity similar to those of the standard antioxidant ascorbic acid [73]. In addition, pomegranate peel extract significantly reduced the pro-oxidative effect of tert-butyl hydroperoxide in a dose-dependent manner and protected against Aβ_42_-induced oxidative stress and ROS generation in HMC3 human microglia—an effect attributed by the authors to PUC polyphenols [75]. Supplementation of 4-month-old AD-mice (APPsw/Tg2576) for 15 months with 4% pomegranate attenuated lipid peroxidation and protein carbonylation, and restored the activities of crucial antioxidant effectors SOD, catalase (CAT), glutathione peroxidase (GPx), Glutathione reductase (GSH) and Glutathione S transferase (GST) in hippocampus and the cerebral cortex [83]. Moreover, supplementation with pomegranate peel extract for 28 days significantly reduced the brain histopathological changes in AlCl_3_-induced AD Sprague Dawley rats, concomitantly with increased levels of antioxidant enzymes and reduced the levels of lipid peroxidation [84]. These results agree with the outcome of our experiments, as PSO pre-treatment prevented the increase or even reduced MDA levels in N2a cells, in the absence or presence of H_2_O_2_ (Figure 4c). In addition, PSO increased SOD1 levels after LPS-induction (Figure 4a,b). We hypothesize this is an effect attributed to the antioxidant properties of the extract, thus reducing free radicals and restricting the need for SOD1 induction.

Furthermore, we additionally performed a preliminary biochemical analysis of sera isolated from MCI patients who consumed PSO for 12 months in addition to adhering to the MeDi. To the best of our knowledge, this is the first time such an intervention has been clinically applied for the selective management of MCI. Individuals who received PSO demonstrated improved total cognition, verbal episodic memory, and processing and executive functions compared to those who did not. The consumption of pomegranate products by animal models of dementia or human patients has been previously associated with enhanced memory performance and improved cognitive function [85]. In a study by Bookheimer et al., 4-week PJ supplementation to middle-aged and older adults reporting memory complaints led to improvements in verbal memory, and increased neural activity during verbal and visual memory tasks [86]. However, in a 12-month randomized clinical trial employing also MCI participants, results suggested that participants consuming PJ did not exhibit statistically significant differences in memory performance. These results indicate that PSO could be a more robust supplementation against cognitive decline, as consumption of this extract not only preserved but also enhanced cognitive functions in MCI patients.

Expanding on the investigation, our biochemical results suggest that the 12-month consumption of PSO had an ameliorative effect on AD hallmark biomarkers, measured in the sera of MCI patients. Notably, MCI patients who had consumed PSO exhibited improvements in Aβ and p-tau-181 levels, as well as in the Aβ_42_/Aβ_40_ and p-tau-181/Aβ_42_ ratios (Figure 5 and Figure 6). Notably, the serum Aβ_42_/Aβ_40_ and p-tau-181/Aβ_42_ ratios are recognized as stronger factors than the levels of Aβ or tau alone and correlate strongly with brain atrophy and disease progression [87,88]. These measurements indicate that 12-month PSO consumption can promote a generalized amelioration in AD molecular pathology, as reflected in critical serum biomarkers.

These positive results align with preclinical studies demonstrating the ability of PSO to mitigate neuroinflammation, reduce oxidative stress, and modulate amyloid and tau pathology [33,89]. In addition, previous in vivo studies in AD animal models have positive results regarding the implication of pomegranate ingredients. Treatment of AD APP/PS1 transgenic mice with PUC improved cognitive behavioral performance, and reduced neuroinflammation, amyloid accumulation, and tau phosphorylation in the brain [90]. Chen et al. have simulated brain aging in vivo employing D-galactose and explored the possible alleviating effects of PUC on learning and memory deficits and hippocampal degeneration. Intragastric administration of PUC for 8 weeks efficiently alleviated cognitive deficits in D-galactose-induced aging mice and prevented neurotoxicity and morphological alterations in hippocampal structure [91].

We observed that MCI patients who consumed PSO for 12 months, exhibited decreased levels of TNF-α (Figure 7a), thus underlying the appeasing nature of pomegranate bioactive compounds in inflammation. Our results come in agreement with the studies by Rojanathammanee et al. and Essa et al., who demonstrated that in vivo pomegranate or PUC and EA supplementation reduced brain pro-inflammatory cytokines (IL-1β, IL-6, and TNF-α) in rodent models of AD [33,92]. In another study, PUC succeeded in reversing neuroinflammation in mice repeatedly injected with LPS to induce cognitive impairment, with authors underlining the significance of NF-κB (p50) inactivation/binding by PUC [93]. An additional statistical outcome of the present study is that the levels of TNF-α correlate significantly with the levels of p-tau181 and the crucial ratio ptau181/Aβ_42_ (Figure 7b,c). This is a conclusion that was also documented in a previous study by our team, with another intervention for MCI patients employing physical education assays, with the ratio p-tau-181/Aβ_42_ significantly correlating with serum IL-1β, IL-6 and TNF-α [49,50]. Thus, the beneficial effects of PSO that have been identified could be at least partially attributed to the anti-inflammatory mechanisms of PSO, making this natural extract a promising candidate for assisting the management of inflammation and neurodegeneration in conditions such as AD and other neuroinflammatory disorders.

## 5. Conclusions

This study presents compelling evidence for the neuroprotective potential of PSO, attributed to its antioxidant, anti-inflammatory, and anti-amyloidogenic properties. By reducing key markers of neuroinflammation (IL-1β, TNF-α), oxidative stress (MDA, iNOS), and neurodegeneration (Aβ_42_, tau, and p-tau181), PSO demonstrates a broad-spectrum therapeutic impact οn both a preclinical model and on patients with mild cognitive impairment. These findings render PSO a promising natural intervention fortifying cognitive health.

## Figures and Tables

**Table 1:** Demographics of MCI patients assigned and non-assigned to PSO intervention.

	**Blood Donor Demographics**		
**Demographics**	**Control**	**PSO**	***p* Values**
**Participants Number (N)**	23	31	
**Gender (Female/Male)**	21/2	19/12	>0.999
**Age (years)**	75.09 ± 7.2	72.81 ± 7.4	0.2637
**Education (years)**	11.83 ± 3.35	11.4 ± 4.1	0.8442
**MMSE**			
**Control Baseline**	**Control After**	**PSO Baseline**	**PSO After**
27.35 ± 1.555	26.96 ± 1.665	27.65 ± 1.380	28.45 ± 1.877 ^a,b^

All values are provided as Mean ± Standard Deviation (SD). Statistical analysis for differences between groups was performed using the Graph Pad Prism 8 statistical package. No statistically significant differences (*p* < 0.05) were found between age, education, and gender between groups. MCI: Mild Cognitive Impairment; PSO: pomegranate seed oil intervention. Statistical analysis for differences between groups was performed using the Graph Pad Prism 8 statistical package. Statistical tests exploited in the study were chosen after performing analyses for the normality of residuals. The statistical significance level was set at *p* ≤ 0.05. ^a^: comparison between PSO Baseline-after (*p* = 0.0046). ^b^: comparison between PSO after-Control After (*p* = 0.0015).

**Table 2:** Biomarkers in the blood serum of MCI patients at the beginning of the study (baseline) and at the end of the study (12 mo.). Levels of Aβ_42_, Aβ_40_, p-tau181, and TNF-α were determined with ELISA. Ratios of Aβ_42_/Aβ_40_, p-tau181 /Aβ_42_ were calculated afterward. All values are provided as Means ± Standard Deviation (SD).

Biomarker	Biomarker Analysis of Study Groups				
	Control (N = 23)		PSO (N = 31)		*p*-Values
	Baseline (B)	12 mo.(A)	Baseline (B)	12 mo.(A)	
**Aβ_42_ (** **pg/mL)**	8.197 ± 2.343	6.157 ± 2.671	8.999 ± 3.280	10.22 ± 2.984	CB-CA:0.0025 PSOB-PSOA: 0.0729 CA-PSOA: <0.0001
**Aβ_40_ (** **pg/mL)**	21.70 ± 3.123	24.15 ± 3.395	20.48 ± 5.628	16.22 ± 3.144	CB-CA: 0.0002 PSOB-PSOA: 0.0014 CA-PSOA: <0.0001
**Aβ_42_/Aβ_40_**	0.3842 ± 0.1140	0.2596 ± 0.1256	0.4602 ± 0.1961	0.5834 ± 0.2285	CB-CA: 0.0005 PSOB-PSOA: 0.0146 CA-PSOA: <0.0001
**p-tau181 (pg/mL)**	11.41 ± 2.515	14.36 ± 2.862	12.13 ± 3.247	7.106 ± 5.485	CB-CA: <0.0001 PSOB-PSOA: 0.0001 CA-PSOA: <0.0001
**ptau-181/** **Aβ_42_**	1.512 ± 0.6242	2.766 ± 1.291	1.504 ± 0.6499	0.7394 ± 0.6782	CB-CA: 0.0004 PSOB-PSOA:0.0004 CA-PSOA: <0.0001
**TNF-α** **(pg/mL)**	32.09 ± 8.332	34.56 ± 8.651	33.41 ± 5.771	27.57 ± 6.098	CB-CA: 0.2747 PSOB-PSOA: 0.0008 CA-PSOA: 0.0010

Statistical analysis for differences between groups was performed using the GraphPad Prism 8 statistical package. A paired *t*-test was applied to determine statistical differences between baseline and 12 mo. values, for each study group. An unpaired *t*-test was performed to compare the effect of the PSO intervention versus the Control group at the end of the study. Statistically significant differences (*p* < 0.05). CB: Control Baseline; CA: Control After; PSOB: PSO Baseline; PSO A: PSO After.

## Data Availability

The datasets generated during and/or analyzed during the current study are available from the corresponding author on reasonable request.

## Supplementary Materials

The following supporting information can be downloaded at: https://www.mdpi.com/article/10.3390/biology14050548/s1. Figure S1: Standard curve for the determination of various concentrations of malondialdehyde (MDA) with thiobarbituric acid (TBA) reaction; Figure S2: Indicative Ponceau S staining of blotted proteins on 0.45 μM nitrocellulose membrane, from N2a lysate samples run under denaturing SDS electrophoresis; Figure S3: Viability assessment of N2a cells exposed to 0–100 μg/mL of pomegranate seed oil (PSO); Figure S4: Cognitive state of the MCI participants of the study, as determined by the mini-mental state examination (MMSE) test; Table S1: List of antibodies and of their dilutions that were employed in Western blotting analyses; Section S1.1: Raw membranes of Western blot with crop points; Section S1.2: Membranes employed in Western blot analysis.
