# Supplementary material for: Neuroprotective, Antioxidant and Anti-Inflammatory Effect of Greek Pomegranate Seed Oil on N2a Neuroblastoma Cells and Mild Cognitive Impairment Patients
Source: Biology (Basel). 2025 May 15;14(5):548. doi: 10.3390/biology14050548 (PMC12109235; doi:10.3390/biology14050548)
Supplement: Supplementary file 1 [file biology-14-00548-s001.zip › biology-3597985-supplementary.pdf]

Supplementary Materials

Index

Content	Page
**Section S1. The Effect of PSO on N2a neuroblastoma cells**	3
**Figure S1.** Standard curve for the determination of various concentrations of malondialdehyde (MDA) with thiobarbituric acid (TBA) reaction.	3
**Figure S2.** Indicative Ponceau S staining of blotted proteins on 0.45 μM nitrocellulose membrane, from N2a lysate samples run under denaturing SDS electrophoresis.	3
**Figure S3.** Viability assessment of N2a cells exposed to 0-100 μg/mL of pomegranate seed oil (PSO).	4
**Table S1.** List of antibodies and of their dilutions that were employed in Western blotting analyses	5
**Section S1.1** Raw membranes of Western blot with crop points	6
**Section S1.2** Membranes employed in Western blot analysis	10
**Section S2. The effect of PSO consumption on MCI patients**	14
**Figure S4.** Cognitive state of the MCI participants of the study, as determined by the mini-mental state examination (MMSE) test	14

Section S1. The Effect of PSO on N2a neuroblastoma cells

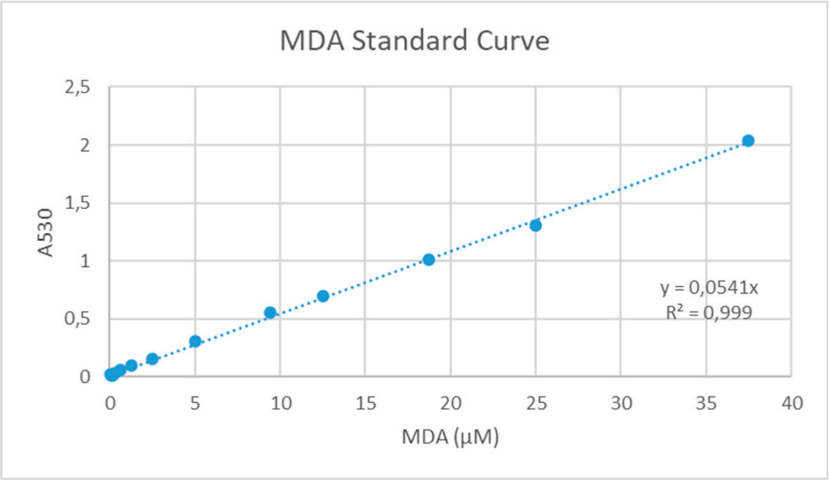

Figure S1. Standard curve for the determination of various concentrations of malondialdehyde (MDA) with thiobarbituric acid (TBA) reaction.

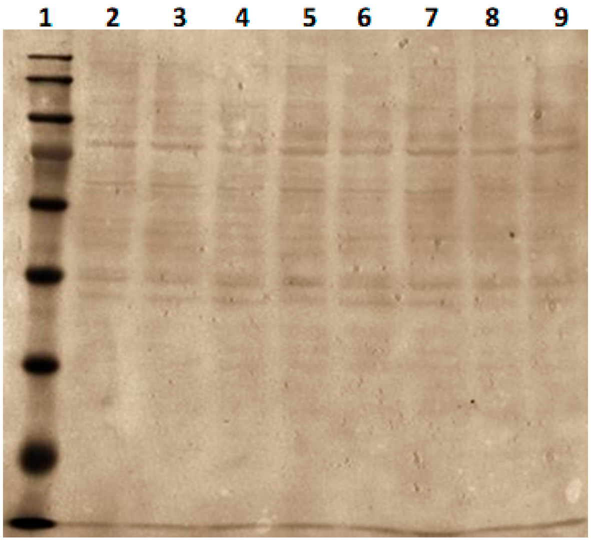

Figure S2. Indicative Ponceau S staining of blotted proteins on 0.45 μM nitrocellulose membrane, from N2a lysate samples run under denaturing SDS electrophoresis. Labels: **1.** Molecular weight protein markers (#MWP03, Nippon Genetics, Japan); **2.** Control; **3.** LPS 1 μg/mL; **4.** PSO 0.2 μg/mL + LPS; **5.** PSO 10 μg/mL + LPS; **6.** PSO 25 μg/mL + LPS; **7.** PSO 0.2 μg/mL; **8.** PSO 10 μg/mL; **9.** PSO 25 μg/mL

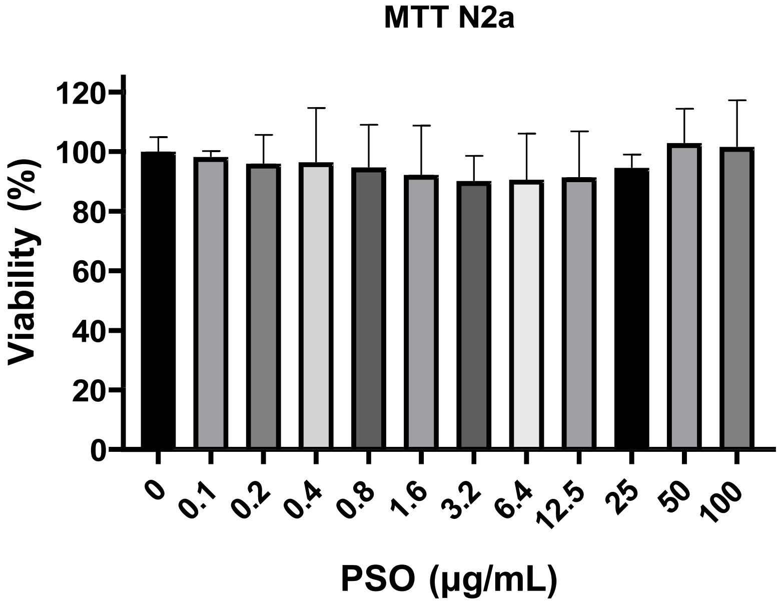

Figure S3. Viability assessment of N2a cells exposed to 0-100 µg/mL of pomegranate seed oil (PSO). Viability has been determined by the MTT assay. Bars represent mean values ± SDs from three independent experiments. Statistical analysis was performed with ordinary one-way ANOVA (without correction for multiple comparisons). No statistically significant differences in viability of N2a cells were verified.

Table S1. List of antibodies and of their dilutions that were employed in Western blotting analyses

Target	Host	Cat. Number	Company	Dilution	Secondary antibody dilution
**APP**	Mouse	60342-1-Ig	Proteintech	1:2000	1:4000*
**Aβ_{42}**	Rabbit	14974	Cell Signaling Technology	1:1000	1:10000#
**tau**	Rabbit	46687	Cell Signaling Technology	1:1000	1:15000#
**p-tau181**	Rabbit	12885	Cell Signaling Technology	1:4000	1:15000#
**iNOS**	Rabbit	18985-1-AP	Proteintech	1:4000	1:20000#
**IL-1β**	Rabbit	L0328Y	Cusabio	1:1000	1:15000#
**TNF-α**	Mouse	sc-52746	Santa Cruz Biotechnology	1:400	1:4000*
**SOD1**	Rabbit	10269-1-AP	Proteintech	1:30000	1:20000#
**β-actin**	Mouse	sc-47778	Santa Cruz Biotechnology	1:1000	1:4000*

***** HRP-conjugated anti-mouse IgG binding protein (#sc-516142, Santa Cruz Biotechnology)

HRP-conjugated goat anti-rabbit IgG antibody (#SA00002-2, Proteintech)

Section S1.1 Raw membranes of Western blot with crop points

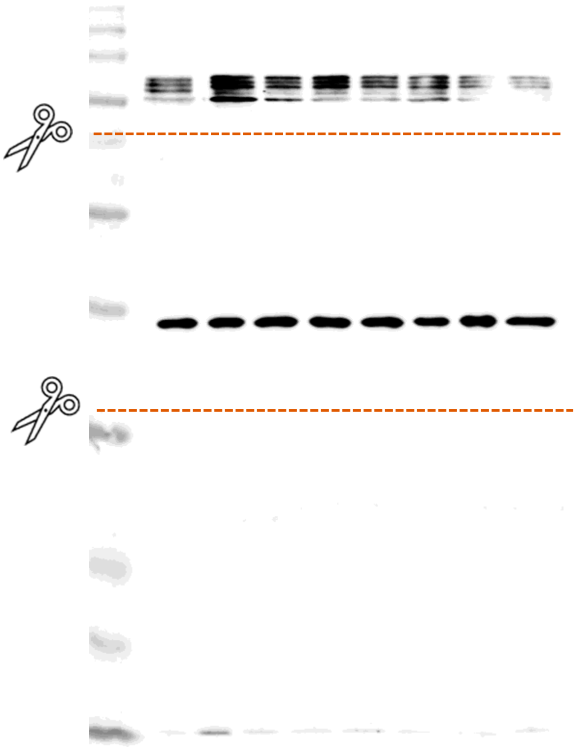

FIGURE 1a

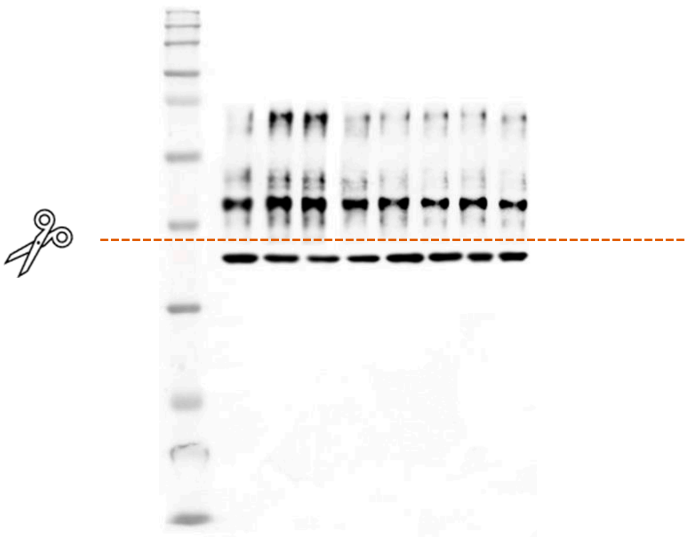

FIGURE 2a

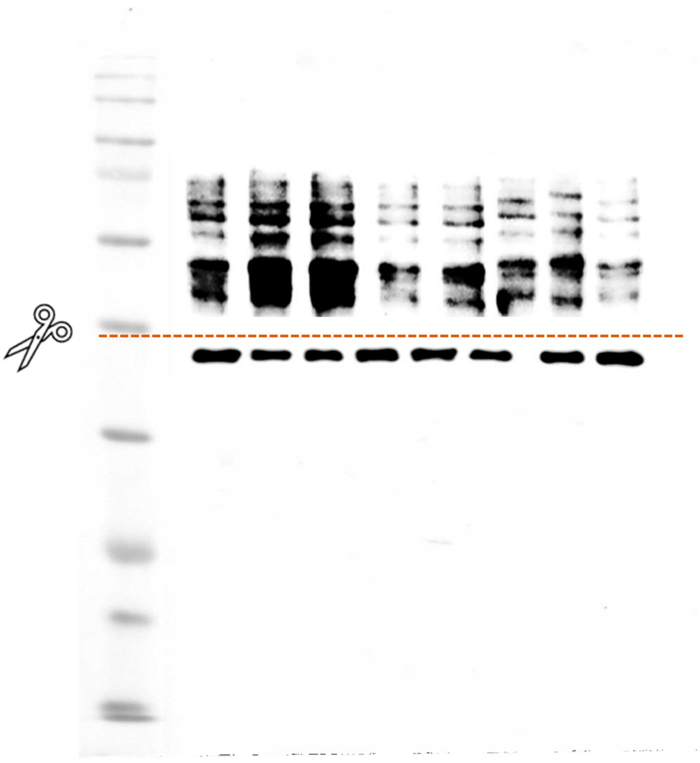

FIGURE 2b

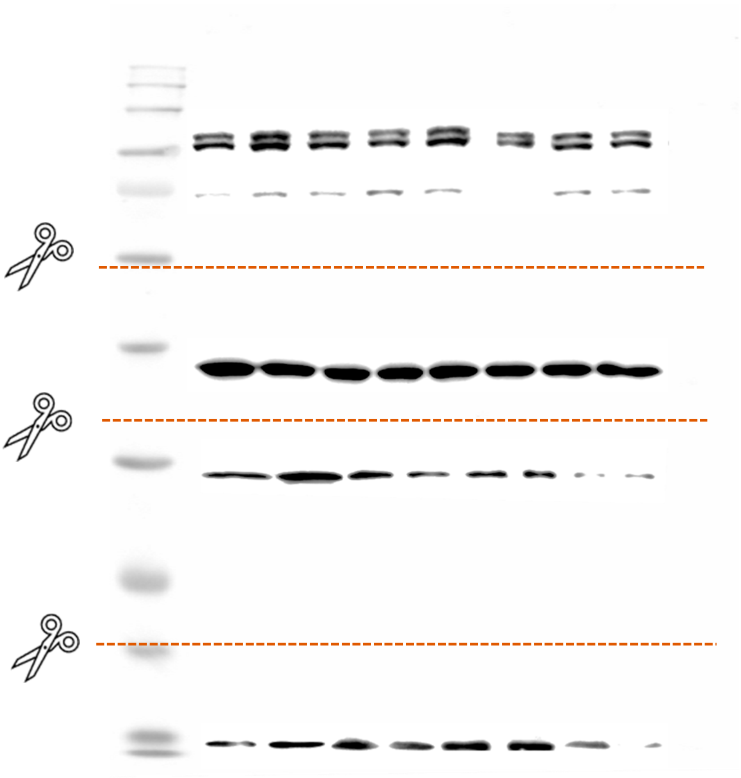

FIGURE 3a

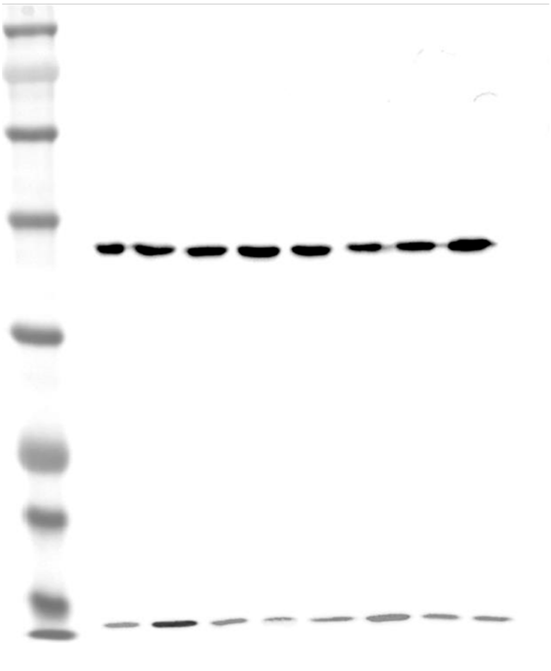

FIGURE 4a

Section S1.2 Replicates employed in the Western Blotting analysis

APP

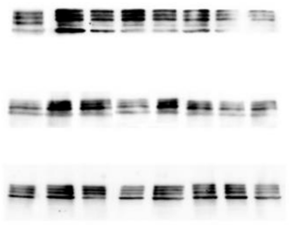

Aβ42

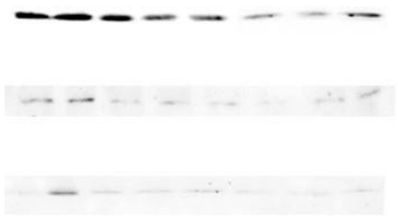

Tau

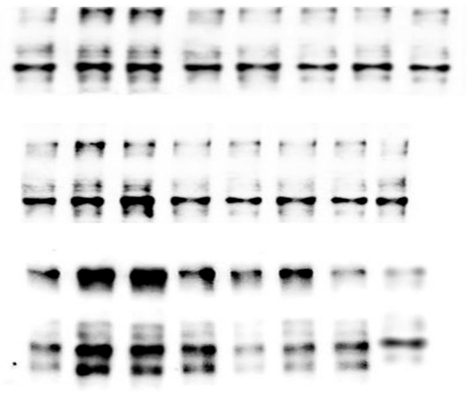

p-tau181

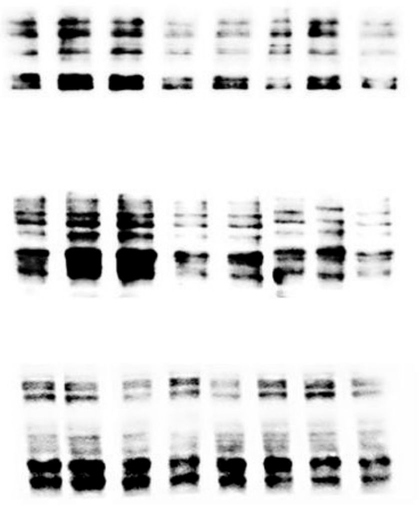

iNOS

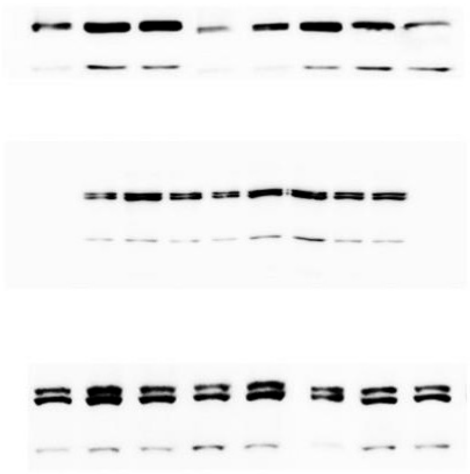

TNF-α

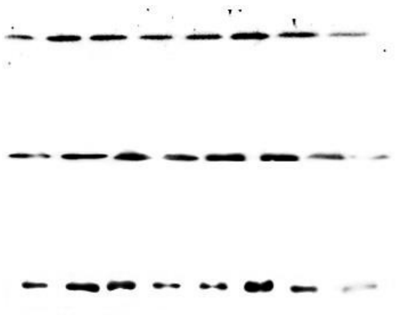

IL1β

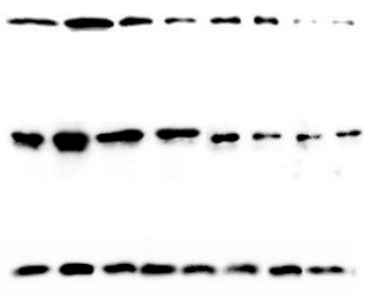

SOD1

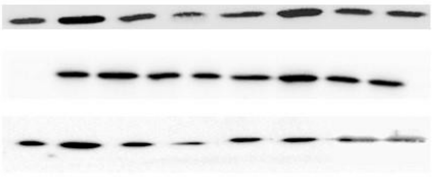

Section S2. The effect of PSO consumption on MCI patients

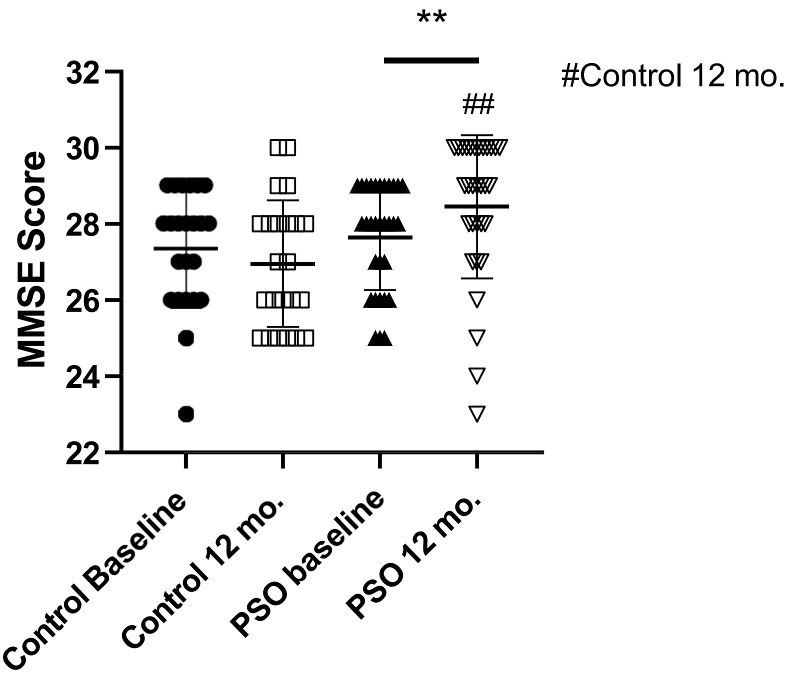

Figure S4. Cognitive state of the MCI participants of the study, as determined by the mini-mental state examination (MMSE) test. Statistical analysis was performed paired t-test between baseline and 12 mo. scores, and with unpaired t-test between control 12 mo. and PSO 12 mo. Statistical significance for $p < 0.05$. ** or ##: $p < 0.01$.
